# Wideband Channel Characterization for 6G Networks in Industrial Environments

**DOI:** 10.3390/s21062015

**Published:** 2021-03-12

**Authors:** Ahmed Al-Saman, Marshed Mohamed, Michael Cheffena, Arild Moldsvor

**Affiliations:** 1 Department of Manufacturing and Civil Engineering, Norwegian University of Science and Technology (NTNU), 2815 Gjøvik, Norway; marshed.mohamed@ntnu.no (M.M.); michael.cheffena@ntnu.no (M.C.); 2 Department of Electronic Systems, Norwegian University of Science and Technology (NTNU), 2815 Gjøvik, Norway; arild.moldsvor@ntnu.no

**Keywords:** millimeter-wave propagation, radio channel, indoor environment, 108 GHz, industrial wideband channel, 5G, 6G, THz band

## Abstract

Wireless data traffic has increased significantly due to the rapid growth of smart terminals and evolving real-time technologies. With the dramatic growth of data traffic, the existing cellular networks including Fifth-Generation (5G) networks cannot fully meet the increasingly rising data rate requirements. The Sixth-Generation (6G) mobile network is expected to achieve the high data rate requirements of new transmission technologies and spectrum. This paper presents the radio channel measurements to study the channel characteristics of 6G networks in the 107–109 GHz band in three different industrial environments. The path loss, K-factor, and time dispersion parameters are investigated. Two popular path loss models for indoor environments, the close-in free space reference distance (CI) and floating intercept (FI), are used to examine the path loss. The mean excess delay (MED) and root mean squared delay spread (RMSDS) are used to investigate the time dispersion of the channel. The path loss results show that the CI and FI models fit the measured data well in all industrial settings with a path loss exponent (PLE) of 1.6–2. The results of the K-factor show that the high value in industrial environments at the sub-6 GHz band still holds well in our measured environments at a high frequency band above 100 GHz. For the time dispersion parameters, it is found that most of the received signal energy falls in the early delay bins. This work represents a first step to establish the feasibility of using 6G networks operating above 100 GHz for industrial applications.

## 1. Introduction

The worldwide marketing of networks of 5G is underway, increasing the catalysts for the next-generation of wireless technologies of 6G due to the ever-increasing demands for massive connectivity to connect millions of people and billions of machines and the emerging class of real-time, interactive applications, such as autonomous vehicles and virtual reality [1,2]. The rapid growth of emerging applications leads to the never-ending growth of mobile data traffic. The global mobile data traffic will reach up to five zettabytes by 2030 according to the International Telecommunication Union’s (ITU) forecast [3]. The direct way to resolve the shortage of the current occupied spectrum of mobile network operators is a spectrum extension to the unoccupied range [4]. The Federal Communications Commission (FCC) has granted the 95 GHz to 3 THz frequency band for 6G research [5,6]. The details of the 6G wireless communication vision, requirements, and applications were provided in [5,7] and some references therein.

The huge available bandwidth in terahertz (THz) frequencies, i.e., 0.1–3 THz, can be used to fulfill the dramatic demands for future data traffic [8]. The higher frequencies are expected to experience high free space, reflection, and scattering losses, which implies that future wireless networks, i.e., 6G, may rely on line-of-sight (LoS) transmission and highly directional steerable antennas with high gains to overcome the high losses [8,9]. Ultra-high-speed and high-efficiency indoor wireless networks are required for the future of wireless connectivity in 6G and beyond. It is also expected that 6G communications will continue to support the applications of industries including the automation of factories [10,11]. In 6G wireless networks, the connection density will increase rapidly and leads to building Industrial Internet of Things (IIoT) networks. Massive industrial devices will be connected in IIoT networks that require an extra-high data rate and low latency [12]. To support the applications of IIoT, the 6G systems will provide a data rate of 1 Tb/s and a latency of 0.1 ms [13]. As the indoor environments, including industrial environments, represent rich sources of scattering objects for radio channel propagation, an extensive THz channel characterization for indoor scenarios is needed to facilitate the design of the infrastructure for 6G and beyond. For this research objective, many academic and research centers have begun to study the THz radio propagation channel for 6G future wireless communications. The relevant state-of-the art for 6G radio propagation channels is discussed in the following section, and Section 3 gives an overview of radio propagation measurements in industrial environments. Then, Section 4, Section 5 and Section 6 describe our measurement campaigns and the results before the conclusion is drawn in Section 7.

## 2. Overview of Radio Wave Propagation for 6G Systems

In this section, previous studies for the radio propagation channel at different candidate frequencies of the 0.1–1 THz band for the future 6G wireless communication system are reviewed.

### 2.1. Weather Effects

In outdoor environments, many studies have been conducted to investigate the effects of weather conditions at different frequencies of the 0.1–1 THz frequency band [14,15,16,17,18,19]. In [14], for outdoor measurement at 200 GHz along an 8 m LoS link, it was observed that the snow attenuation was approximately 2 dB. At a 0.4 km path length, the LoS measurements at 103 GHz and 120 GHz in [15,16,17] showed that the rain attenuation is less than 5 dB at a low rainfall rate below 10 mm/h and less than 20 dB at a high rainfall rate of 100 mm/h. Based on the conducted measurements along an 800 m LoS link at 140.7 GHz in [18], it was found that the rain attenuation is less than 8 dB and 20 dB at rainfall rate of 10 mm/h and 100/h, respectively. In [19], for the conducted measurements at 148 GHz and 156 GHz along a 325 m LoS link, it was reported that the rain attenuation is below 6 dB at a rainfall rate below 40 mm/h.

### 2.2. Propagation Mechanism

The wireless communication systems at a frequency band above 100 GHz are expected to be strongly influenced by the variations of the reflective angle and the scattering of different stratified materials over the frequency and incident angle, as they will affect both LoS and NLoS propagation [20]. Hence, the impacts of reflections and scattering from different materials in radio propagation above 100 GHz have gained attention from different research groups in the literature [20,21,22,23,24]. In [20], the reflection terahertz time domain spectroscopy measurements and matching transfer matrix simulations of the frequency dependent reflection coefficient of multilayer building materials were presented for a set of angles in the 100–500 GHz frequency band, where a plaster sample, covered with white paint, and a double pane glass window were studied as examples for two commonly encountered multilayer structures in indoor scenarios. Based on the simulations performed over frequencies from 1 GHz to 1 THz in [24] using three different materials with incident angles of 10${}^{\circ }$–90${}^{\circ }$, it was shown that the received scattered power increases as the frequency increases, and the scattered power falls off sharply when the incident wave becomes grazing and most of the incident power is reflected. In [21], the THz links’ performance was studied in an indoor environment with different surface reflections (wall, ceiling, and furniture) based on narrowband measurements at different frequencies in the range of 0.1–1 THz (100, 200, 300, 400, and 625 GHz). The reflection and scattering from different materials, glass, plastic, hardwood, concrete, and aluminum, were also investigated in typical indoor office environments [22] based on time domain spectroscopy measurements in the 0.3–3 THz band and ray-tracing simulation. Considering the reflections from the walls, floor, and ceiling and randomly picked reflection points, simple deterministic propagation models were derived in [23] for LoS and NLoS indoor environments. In [25], the effective attenuation was measured at 100 GHz for typical building materials such as wood, tiles, concrete bricks, and a gypsum plate, and it was observed that the effective attenuation of most of the building materials is polarization sensitive. Jacob et al. [26] studied the diffraction from objects, such as wedges, edges, and cylinders, including the human body and different materials, such as metal and the wood knife-edge model, and reported that the best agreements were found between the uniform geometrical theory of diffraction and the measurement results at 300 GHz in indoor environments. However, it was also concluded that the diffraction from edges or wedges can be neglected almost everywhere in a room scenario using ray-tracing tools [26].

### 2.3. Indoor Channel Characteristics

The work in [9,27,28,29,30,31] conducted wideband measurements at 300 GHz in different indoor environments using a vector network analyzer (VNA)-based sounder. The path loss, root mean squared delay spread (RMSDS), and absorption attenuation were investigated at 300 GHz in short-range desktop and indoor office LoS and non-LoS (NLoS) scenarios in [9,27]. These studies observed 3.9 dB misalignment losses for a 1.67 m LoS link. However, for NLoS links at a 2.12 m to 4.77 m distance, References [9,27] reported additional losses of 12.9–30 dB and 29.7–52.7 dB for one and two reflections, respectively, depending on the involved surfaces of reflective objects. They also showed that about 86.7 dB and 65.5 dB absorption attenuations resulted at 300 GHz along a 0.1 m link from obstructions of the ray path by a 3.5 cm thick fiberboard door and a 2.5 cm thick window, respectively. In [28,29], ultra-wideband measurements were carried out in a small indoor office at 300 GHz with approximately a 50 GHz bandwidth using virtual antenna arrays, and the path loss, reflection loss, K-factor, RMSDS, and capacity were investigated at different angles-of-arrival/angles-of-departure (AoAs/AoDs). The measurements showed about a 12–15 dB loss per reflection, and the RMSDS ranged from 1.6 to 3.7 ns, while the AOA angular spread ranged from 15.7${}^{\circ }$ to 40.1${}^{\circ }$ for different Tx locations [28]. A stochastic spatio-temporal indoor channel model at 300 GHz was introduced in [29] to provide complete channel characteristics in an indoor office scenario. Kim and Alenka [30] presented an ultra-wideband statistical characterization at 300 to 320 GHz along a 0.7 m LoS link and a 0.3 m NLoS link. Three different materials were used to completely block the LoS signal from Tx to Rx on a desktop in a typical indoor environment. This study found that the path loss exponent (PLE) for the LoS link was around 1.9 and that the mean value of the RMSDS was 428.4 ps for the LoS link, while the RMSDS values for the NLoS link varied from 187 ps to 227 ps based on the obstacles’ materials. The same value of the PLE was reported at 300–316 GHz for an indoor LoS channel in [31] based on wideband channel measurement along a 0.25 m LoS link. In [32], wideband LoS and NLoS indoor measurements were conducted using a VNA-based system in the 260–400 GHz frequency band to investigate the path loss, angle-of-arrival (AoA), and absorption coefficients of different scattering objects.

Measurements at 140 GHz have been conducted in different indoor environments [8,31,33,34]. The path loss, reflection, and RMSDS were investigated at 140 GHz in [33] based on ultra-wideband VNA-based system measurements of a 60 GHz bandwidth in LoS, obstructed LoS (OLoS), and reflected NLoS indoor environments over 0.4 to 0.9 m links. It was shown that the PLE is around 1.97 for the LoS and the PLEs are 1.89, 2.13, and 2.95 for the OLoS using plastic, glass, and ceramic obstructions between Tx and Rx, respectively. For reflected NLoS using an aluminum plate reflector, the results in [33] showed that the measured path loss was very similar to the free space path loss (FSPL). A similar PLE around 2.22 was reported for the LoS in an indoor shopping mall [34] based on wideband measurements of the 4 GHz bandwidth using a VNA system at 140 GHz along a 35 m link. The PLE in an indoor office environment at 140 GHz with a 1 GHz bandwidth was 2.02 using correlation-based sounding measurements [8]. Based on all mentioned radio propagation studies at 140 GHz, it can be noted that the PLE for the measured signal was close to a free space PLE of two. It was also found that the PLE for the 126–156 GHz measured band was 1.9 (close to a free space PLE of two) based on wideband measurements using a VNA in an indoor office along a 1.6 m LoS link [35]. At three different bands of 30, 140, and 300 GHz, the close-in free space reference distance (CI) model, floating-intercept (FI) model, alpha-beta-gamma (ABG) model, and close-in frequency dependent (CIF) model were investigated, and the results showed that the ABG and CIF models were more stable than the CI and FI models [31].

### 2.4. Throughput in 6G Networks

In [36], the channel was characterized at 350 GHz based on ray-tracing simulations where the high-gain antennas influence the transmission conditions and position of the transmitter (Tx) for optimal signal coverage; and the achievable data rate was presented for different propagation conditions in generic indoor scenarios. At the same frequency of 350 GHz, Bhardwaj et al. [37] measured the propagation loss using a VNA-based system to estimate the communication link performance and stated that the wireless communication link at 350 GHz can support 1 Gpbs and 100 Gbps along 8.5 m and 1 m links, respectively. In [32] it was reported that 1.3 Tbps and 0.9 Tbps throughput can be achieved over 0.1 m links within a 140 GHz bandwidth of the 260–400 GHz band for LoS and NLoS scenarios, respectively. Based on the applied full 3D ray tracing tool in an indoor office for a THz link of 5.5 m at 300 GHz, it was found, using highly directive antennas, that the THz channels can support up to several 100 GSymbols/s without any inter-symbol interference (ISI) [38].

A summary of these channel characterizations at 0.1–1 THz is provided in Table 1.

## 3. Overview of Radio Propagation Measurements in Industrial Environments

The radio channel propagation characteristics in industrial environments are quite different from typical indoor office environments due to the infrastructure of the industrial environments, which contain many electrical and mechanical machines [39]. The rich scattering in industrial environments due to the highly reflective structures produces heavy multipath propagation channels, especially at high frequencies such as millimeter-wave (mmWave) and THz [40].

Many radio propagation studies have been conducted in different indoor industrial environments in the sub-10 GHz band. Ultra-wideband measurements were carried out in two different industrial environments at 3.1–5.3 GHz and 3.1–10.6 GHz frequency bands [41]. It was found that the PLE was 1.1 along a 16 m point-to-point NLoS link, and the RMSDS values ranged from 28 to 38 ns and from 34 to 51 ns for LoS and NLoS, respectively. In [42], the path loss and K-factor characteristics at three different frequencies (0.9, 2.4, and 5.2 GHz) were presented based on narrowband measurements along 15 to 140 m Tx-Rx separation distances in four factories with different topographies. Different PLEs for each frequency were reported, ranging from 0.87 to 4.47 and from 1.68 to 4.49 for non-fixed (FI model) and fixed intercept (CI model) path loss models, respectively, in different facilities for wood and metal material processing. Detailed radio propagation characteristics for radio channels such as path loss, RMSDS, and K-factor at 2.2 and 5.4 GHz in various industrial environments were provided in [43]. A comprehensive review of radio channel measurements in different industrial environments in sub-6 GHz band was presented in [39].

Radio propagation measurements at the sub-THz band in indoor industrial environments are expected to attract more research interest [40]. For instance, a few mmWave radio propagation measurements have been conducted in various industrial environments at 28 GHz [44,45,46,47,48,49,50] and 60 GHz [44,51,52]. Narrowband measurements were conducted inside a factory environment at 28 GHz in [46] to study the path loss at a 5–100 m distance over 400 LoS and NLoS measured points, and it was shown that the PLE values were 2 and 4.1 for LoS and NLoS, respectively. Based on the conducted wideband measurements at 28 GHz in an engineering workshop, it was observed that the reflected and refracted mmWave propagation waves from metallic surfaces were significantly high [47]. In [49], based on wideband time domain measurements at 28 GHz with a 2 GHz bandwidth inside a circle-shaped machine hall, the path loss and RMSDS were investigated. It was found that the PLEs of the FI model were 1.9 and 0.9 for LoS and NLoS, respectively, and 1.8 and 2 based on the CI model for LoS and NLoS, respectively. It was shown that the FI model did not fit the measured path loss for the NLoS scenario. Using the same measurements system, the measurements were carried out at 3.7 and 28 GHz in an indoor factory with Tx-Rx separation distances of 4 to 26 m and 9 to 25 m for LoS and NLoS, respectively [50]. Using the ABG path loss model, the PLEs were 2.27 and 3.02 for LoS and NLoS, respectively. The RMSDS values at 28 GHz were 23.1 and 33.6 ns for LoS and NLoS, respectively.

The path loss and RMSDS were studied in [51] based on the conducted wideband measurements using a VNA in a metal cabinet (to emulate the industrial machine) in the frequency band of 57–62 GHz. This study stated that the path loss was low as the PLEs varied from 0.004–0.021 along a Tx-Rx separation distance of 0.25–2.5 m. It was also indicated that the RMSDS values (up to 160 ns) were significantly larger than for typical indoor environments. In [44], the mmWave channel characteristics were investigated at 28 and 60 GHz based on wideband measurements using a VNA in two different factories representing light and heavy industry. The PLE values were 1.98 and 2.15 at 28 GHz for light and heavy industry, respectively, along a 35 m LoS Tx-Rx separation distance. For NLoS scenario at 28 GHz, the PLEs were 2.3 and 5.3 for light and heavy industry, respectively. At 60 GHz, the PLEs were 2.1 (light industry), 1.9 (heavy industry) and 2.4 (light industry), 5.7 (heavy industry) for the LoS and NLoS scenarios, respectively. For LoS scenarios, the mean RMSDSs at 28 GHz and 60 GHz were almost identical with values 13.7 ns (at 28 GHz) and 13.4 ns (at 60 GHz) for light industry and 38.5 ns (at 28 GHz) and 38.3 ns (at 60 GHz) for heavy industry. For NLoS scenarios, the RMSDS values were also almost identical at 28 GHz and 60 GHz with values of 29.1 ns (at 28 GHz) and 29.3 ns (at 60 GHz) for light industry and 49.4 ns (at 28 GHz and 60 GHz) for heavy industry. The physical layer channel characteristics like the multipath delay profile and the link-layer parameters like throughput were investigated based on the conducted measurements at 60 GHz in different industrial sites [52]. The reported studies of mmWave channel measurements in industrial indoor environments are summarized in Table 2.

After a thorough literature review, it can be found that measurements of radio channels in industrial environment are very limited, particularly at high frequencies. In various industrial settings, there is an absolute need for more measurements to understand the wave propagation mechanism accurately. The radio channel characterizations in different indoor industrial environments need more studies at different candidate frequencies in the sub-THz band as it is the promising spectrum for 5G, 6G, and beyond. To the authors best knowledge, industrial radio channel propagation studies at frequency bands above 100 GHz are lacking in the literature.

This paper presents a study of wideband channel characteristics in three different industrial environments in the 107–109 GHz frequency band. The path loss models and the K-factor are investigated. The temporal characteristics are analyzed with respect to various metrics, like the power delay profile (PDP), mean excess delay (MED), and RMSDS.

## 4. Measurement Campaigns

The measurements were conducted using a commercial VNA, R&S ZVA 67 [53], and up-converters, R&S ZVA-Z110 [54]. The up-converters not only convert the signal from 18 GHz to 108 GHz, but also amplify the signal so that no additional amplifiers are required. The setup was calibrated using through-offset-short-match (TOSM) to remove the effects from the connectors, cables, and equipment. The setup resulted in a noise floor of −100 dBm with an intermediate frequency (IF) of 10 MHz.

**Figure 1:**
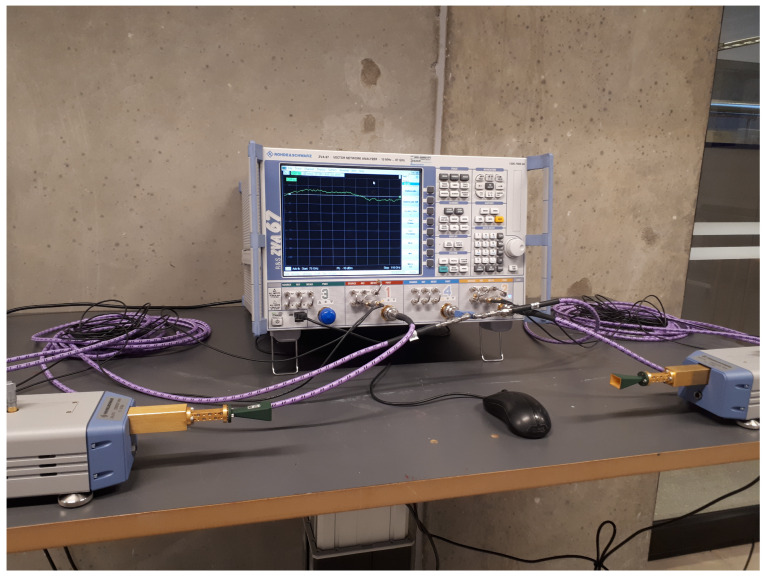
Measurement equipment setup photo to show the cable connection to the VNA and the frequency converters.

Horn antennas were connected to the up- and down-converters with one acting as a transmitter (Tx) and the other as a receiver (Rx). A photo of the measurement equipment and the connections is shown in Figure 1. Both antennas were set at a height of 1.6 m from the floor, with a gain of 21.14 dBi and an azimuth half power beam-width (HPBW) of $15.7^{\circ }$ at the measurement frequency of 108 GHz [55]. To cover a wider angle, the measurements were repeated with antennas set at azimuth angles of $0^{\circ }$, $\pm 15^{\circ }$, $\pm 30^{\circ }$, $\pm 45^{\circ }$, and $\pm 60^{\circ }$. This procedure expanded the beam-width of the antennas to $137.5^{\circ }$ ($120^{\circ }+15.7^{\circ }$), enabling the capturing of more environmental effects of the channel.

**Figure 2:**
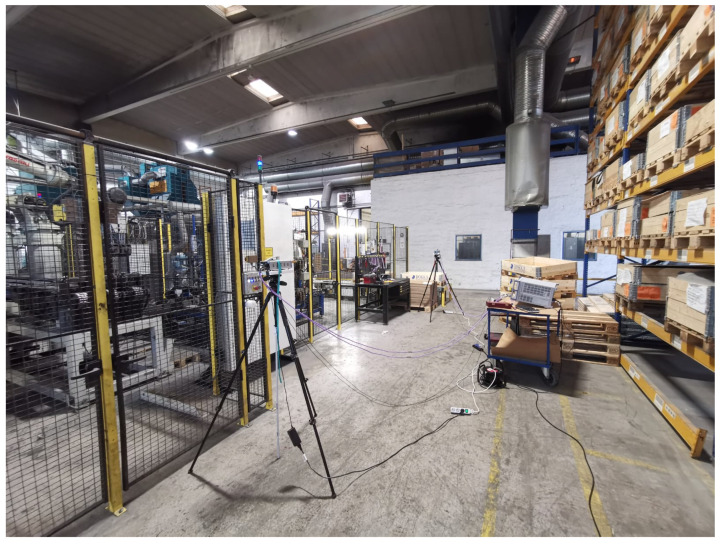
Measurement setup in Manufacturing hall at Dokka Fasteners AS. On the right side, the metallic shelves contain many pieces of heavy-duty equipment inside the wooden boxes. The left side contains many robotic types of machinery surrounded by a metal fence with an metallic alarm box and a grille door.

**Figure 3:**
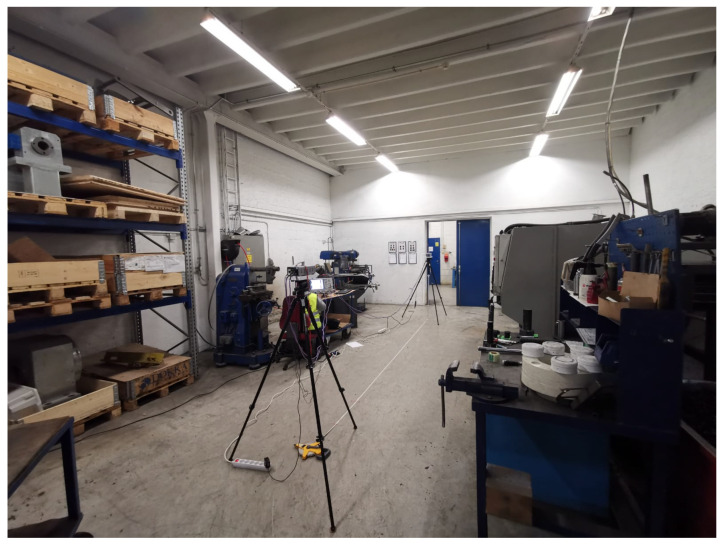
Measurement setup in the Test room at Dokka Fasteners AS. Two big metallic machines are located on the right side. Metallic shelves with may pieces of heavy-duty equipment inside the wooden boxes and two metallic machines on the left side.

**Figure 4:**
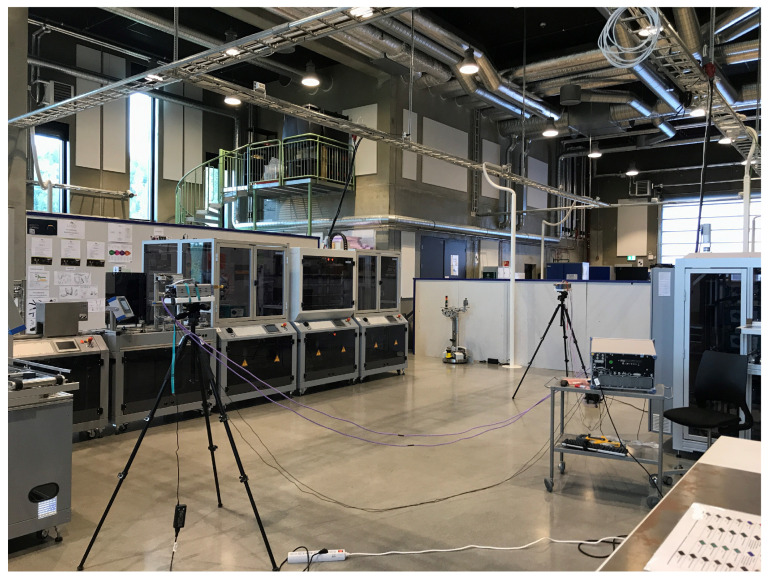
Measurement setup in the Manufacturing laboratory (MANULAB)at the Norwegian University of Science and Technology (NTNU). It contains many pieces of medium size equipment.

The VNA was configured to measure the channel transfer function $S_{21}$ in the frequency domain from 107 to 109 GHz, i.e., central frequency of 108 GHz and span of 2 GHz with 2001 frequency points (a step size of 1 MHz). LoS measurements for distance values ranging from 0.5 to 5 m with an increment of 0.5 m were conducted. The measurement setup parameters are listed in Table 3. The measurements were repeated in three different locations, namely:

- Manufacturing hall at Dokka Fasteners AS. This is a big hall that contains many pieces of heavy duty equipment. The measurements were carried out in a particular place inside the hall between two closed sides as shown in Figure 2. One side was enclosed in metallic shelves containing many pieces of heavy-duty equipment inside the wooden boxes. The other side contained many robotic types of machinery surrounded by a metal fence with an alarm metallic box and a grille door that was closed during the measurements, as shown in Figure 2.
- Test room at Dokka Fasteners AS, which contains a few pieces of heavy duty equipment, as shown in Figure 3. The room contained two big metallic machines on the right side. On the left side, there were metallic shelves containing many pieces of heavy-duty equipment inside the wooden boxes and two metallic machines, as shown in Figure 3.
- Manufacturing laboratory (MANULAB) at the Norwegian University of Science and Technology (NTNU). It contains many pieces of medium size equipment, as shown in Figure 4.

## 5. Post-Processing and Parameters Extractions

### 5.1. Power Delay Profile

The obtained S_21_ from the VNA measurements represents the channel transfer function H(f). The inverse discrete Fourier transform (IDFT) of H(f) represents the channel impulse response |h(τ)|, and ${\left|h\left(\tau \right)\right|}^{2}$ represents the power delay profile (PDP), which shows the multipath components (MPCs) with a resolution limited by the total measured frequency span. To suppress the undesired effect of side lobes when performing the IDFT, a Hanning window ($h_{w}$) was used in the frequency domain. Other windows like Blackman and Hamming were also tested; however, a Hanning window, which is considered a good compromise between low side lobes and reduced resolution, fit the data well and significantly reduced the interfering effect between the MPCs. The mathematical expression for extracting the PDP from the measured S_21_ from the number of frequency samples (*N*) is:

(1) $$\mathrm{PDP}\left(\tau \right)={\left|\frac{1}{N}\sum_{n=1}^{N}\left[H\left(f\right)\times h_{w}\right]\exp\left(j2\pi f_{n}\tau \right)\right|}^{2}$$

**Figure 5:**
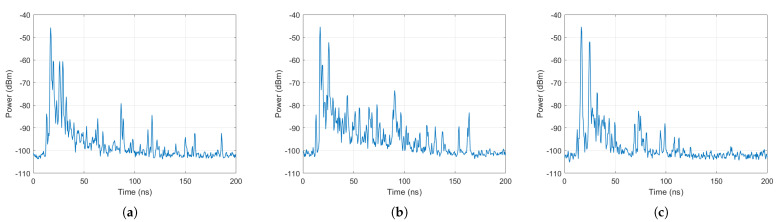
Power delay profile at a 5 m Tx-Rx separation distance in (**a**) the Manufacturing hall at Dokka Fastners AS, (**b**) the Test room at Dokka Fastners AS, and (**c**) NTNU MANULAB.

**Figure 6:**
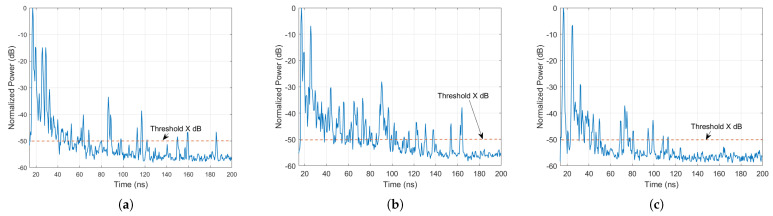
Normalized power delay profile at a 5 m Tx-Rx separation distance in (**a**) the Manufacturing hall at Dokka Fastners AS, (**b**) the Test room at Dokka Fastners AS, and (**c**) NTNU MANULAB.

As mentioned in the measurement setup, horn antennas were used at the Tx and Rx; hence, to capture more environmental effects of the channel, the Tx and Rx antennas were rotated to different angles with a step size of 15${}^{\circ }$ at each Tx-Rx separation distance. The PDP that represents all the received power from all angles at Tx ($A_{T}$) and Rx ($A_{R}$) can be defined as [56]:

(2) $$\mathrm{PDP}\left(\tau \right)=\max_{i,j}{\left|h_{A_{T_{i}},A_{R_{j}}}\left(\tau \right)\right|}^{2}$$

where *i* and *j* are the indices of the rotation angles at Tx and Rx, respectively. Figure 5a–c show the measured PDP among all angles based on (2) at the maximum Tx-Rx separation distance of 5 m in the three testbed industrial environments. The PDP was normalized to 0 dB relative to the maximum power, which was the power of the LoS path in our measurements. Figure 6a–c show the normalized values of the PDP mentioned in Section 5.4.

### 5.2. Path Loss, Received Power, and K-Factor

The path loss (PL) can be calculated from the total sum of the squared amplitude of the measured multipath $p_{l}^{2}$ and the antenna gains as [57]:

(3) $$PL\left(\mathrm{dB}\right)=-10\;\log_{10}\left(\frac{\sum_{l=1}^{L}p_{l}^{2}}{G_{t}G_{r}}\right)$$

where *L* is the total number of measured multipaths and $G_{t}$ and $G_{r}$ are the gains of the Tx and Rx antennas at the center frequency, respectively. Then, the received power can be calculated as:

(4) $$P_{r}\left(\mathrm{dBm}\right)=P_{t}\left(\mathrm{dBm}\right)-PL$$

where $P_{t}$ is the transmitted power set at 0 dBm for all measurements. The K-factor is the ratio of the energy of the dominant path ($E_{d}$), which is the LoS path in our measurements, and the energy of the reflected and scattered paths ($E_{Rs}$) and can be defined as:

(5) $$K\left[\mathrm{dB}\right]=10\;\log_{10}\left(\frac{E_{d}}{E_{Rs}}\right)$$

### 5.3. Path Loss Models

Path loss characterization using empirical models with parameters adjusted according to the measurement scenario is a useful approach in planning engineering. Several models have been proposed in the literature to describe the dependency of the median path loss on the frequency and distance in typical indoor scenarios. In this study, the most commonly used models were considered: the log-distance CI model and the FI model. The CI path loss model can be expressed as [58]:

(6) $${PL}^{CI}\left(f,d\right)\left[\mathrm{dB}\right]=PL\left(f,d_{0}\right)+10n\log_{10}\left(\frac{d}{d_{0}}\right)+X_{\sigma },$$

where $PL\left(f,d\right)$ is the path loss at different frequencies with various Tx-Rx separation distances (*d* in meters), $PL(f,d_{0})$ is the path loss at close-in distance $d_{0}$ of 1 m or less in dB , *n* denotes the distance dependency of path loss, which is the PLE, and $X_{\sigma }$ is a zero-mean Gaussian distributed random variable with a standard deviation of σ dB (shadowing effects). The FI path loss model is defined as [58]:

(7) $${PL}^{FI}\left(d\right)\left[\mathrm{dB}\right]=\alpha +10\beta \log_{10}\left(d\right)+{X_{\sigma }}^{FI},$$

where α and β are the floating-intercept in dB and the slope of the line, respectively. The shadow fading is represented by zero mean Gaussian random variable ${X_{\sigma }}^{FI}$ dB with a standard deviation of σ dB.

### 5.4. Time Dispersion Parameters

All-time dispersion parameters were measured relative to the time of arrival of the first multipath component (the LoS component in the LoS scenario). The PDP was normalized, and all the signals above a *X* dB threshold, where *X* is set to 50 dB, were all considered MPCs. Examples of the normalized PDP at the maximum Tx-Rx separation distance of 5 m in the three industrial environments are shown in Figure 6a–c.

The time dispersion of ultra-wideband signals can be measured from the ratio of the mean excess delay (MED)-to-the RMS delay spread (RMSDS). Here, this ratio represents the spreading factor and is defined as [58]:

(8) $$\mathrm{SF}=\frac{\tau_{MED}}{\tau_{RMSD}}$$

$\tau_{MED}$ and $\tau_{RMSD}$ are the MED and RMSD and can be calculated using (9) and (10), respectively.

(9) $$\tau_{MED}=\frac{\sum_{l}P\left(\tau_{l}\right)\tau_{l}}{\sum_{l}P\left(\tau_{l}\right)},$$

(10) $$\tau_{RMSD}=\sqrt{\bar{\tau^{2}}-{\left(\tau_{MED}\right)}^{2}},$$

where:

(11) $$\bar{\tau^{2}}=\frac{\sum_{l}P\left(\tau_{l}\right)\tau_{l}^{2}}{\sum_{l}P\left(\tau_{l}\right)},$$

$\bar{\tau^{2}}$ is the second central moment of the PDP, and $P(\tau_{l})$ is the received power at the *l*th multipath. The RMSDS provides a measure of the variability of the mean delay, and it is inversely proportional to the coherence bandwidth, which indicates the frequency selectivity of a channel and corresponds to the maximum channel bandwidth that can be used without the production of large ISI at the Rx. The estimation of this factor is important because the duration of each symbol or cyclic prefix for orthogonal frequency division multiplexing (OFDM) modulation must be considerably longer than the RMS delay spread to prevent ISI at the Rx.

## 6. Measurement Results and Analysis

### 6.1. Received Power

**Figure 7:**
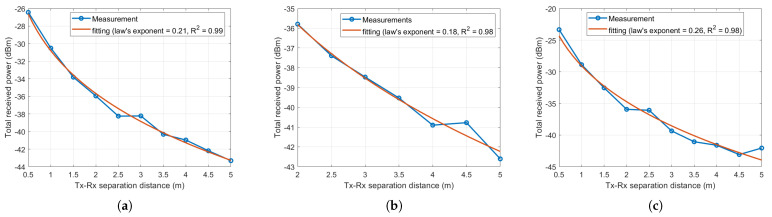
Total measured received power with Tx-Rx separation distance in (**a**) the Manufacturing hall at Dokka Fastners AS, (**b**) the Test room at Dokka Fastners AS, and (**c**) NTNU MANULAB.

Figure 7a–c show the measured received power (the antenna gains are included) with Tx-Rx separation distance. As expected, it is shown that the received power decreases as the Tx-Rx separation distance increases. The received power decays follow the power-law distribution with the law exponents of 0.21, 0.18, and 0.26 for the Manufacturing hall at Dokka Fastners AS, the Test room at Dokka Fastners AS, and NTNU MANULAB industrial environments, respectively. The received power degradation values are around 17 dB and 20 dB at a 5 m Tx-Rx separation distance compared to the received power at 0.5 m for the Manufacturing hall at Dokka Fastners AS and NTNU MANULAB industrial environments, as can be seen in Figure 7a,c, respectively. For the Test room at Dokka Fastners AS industrial environment, the received power degradation is around 7 dB at a 5 m Tx-Rx separation distance compared to the received power at 2 m, as depicted in Figure 7b. However, this is approximately the same power degradation as seen for the two former locations when calculated from 2 to 5 m instead of from a 0.5 to 5 m distance.

### 6.2. Path Loss

**Figure 8:**
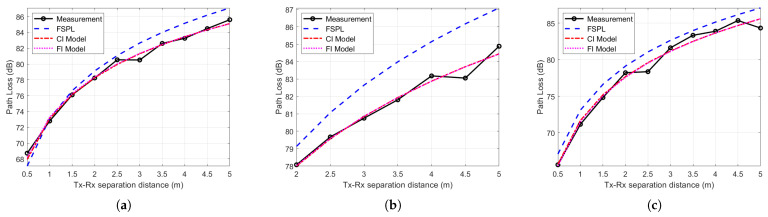
Path loss models in (**a**) the Manufacturing hall at Dokka Fastners AS, (**b**) the Test room at Dokka Fastners AS, and (**c**) NTNU MANULAB. FSPL, free space path loss; CI, close-in free space reference distance; FI, floating intercept.

The CI and FI path loss models are used to investigate the measured path loss at the three locations and compared with the FSP,L as shown in Figure 8a–c. It can be seen from the figures that the CI and FI path loss models fit the measured path loss very well for all environments. It can be noticed that the measured path loss is less than the FSPL with all Tx-Rx separation distances at the NTNU MANULAB and Manufacturing hall at Dokka Fastners AS and for a 2–5 m Tx-Rx separation distance in the Test room at Dokka Fastners AS, implying that the MPCs added up, constructively contributing additional power to the LoS component. The highest measured path loss is 85 dB for all environments at a 4.5–5 m Tx-Rx separation distance. The parameters for the CI and FI path loss models are listed in Table 4. Using reference points ($d_{0}$) of 0.85 m, 1 m, and 1 m, the PLE values for the CI path loss model are 1.7, 1.6, and 1.9 with standard deviations of 0.7 dB, 0.4 dB, and 0.3 dB for the Manufacturing hall at Dokka Fastners AS, the Test room at Dokka Fastners AS, and NTNU MANULAB, respectively. For the FI path loss model, with a floating α of 73.3 dB, 73.1 dB, and 71.5 dB, the slope lines β are 1.7, 1.6, and 2.0 with standard deviations of 0.4 dB, 0.3 dB, and 0.7 dB, for the Manufacturing hall at Dokka Fastners AS, the Test room at Dokka Fastners AS, and NTNU MANULAB, respectively. The values of these parameters of CI and FI along three indoor industrial settings indicate that the CI and FI path loss models have the same performance of accuracy and stability to perform the best fitting for the measured path loss in indoor industrial environments.

### 6.3. K-Factor

**Figure 9:**
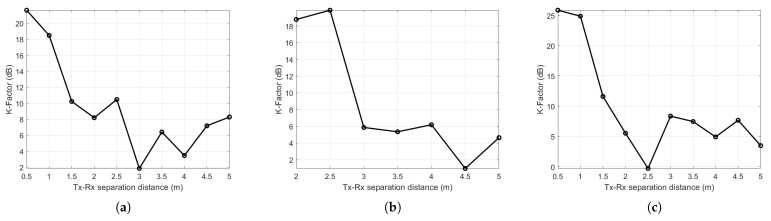
K-factor along with a measurement distance of 5 m in (**a**) the Manufacturing hall at Dokka Fastners AS, (**b**) Test room at Dokka Fastners AS, and (**c**) NTNU MANULAB.

Figure 9a–c present the K-factor plots against the Tx and Rx separation distance at the Manufacturing hall at Dokka Fastners AS, the Test room at Dokka Fastners AS, and the NTNU MANULAB environments, respectively. It is shown that the K-factor varies from 1.9 dB to 21.6 dB, from 0.9 dB to 19.9 dB, and from −0.3 dB to 26 dB at the Manufacturing hall at Dokka Fastners AS, the Test room at Dokka Fastners AS, and the NTNU MANULAB environments, respectively. It can be observed that the K-factor is particularly high when the Tx-Rx separation distance is small. This implies that when the Tx-Rx separation distance increases, the LoS signal component becomes weak. However, it is accordingly clear that this decreasing trend of the K-factor is not monotonic with the distance, which means that with strong NLoS MPCs, the K-factor becomes small regardless of the Tx-Rx separation distance. The K-factor in dB becomes smaller than 0 dB when the total power acquired from NLoS MPCs is greater than the received power of the LoS component that can be observed at a 2.5 m Tx-Rx separation distance at the NTNU MANULAB environment, as shown in Figure 9c. The minimum, maximum, and average values of K-factor are listed in Table 5.

### 6.4. Time Dispersion

**Figure 10:**
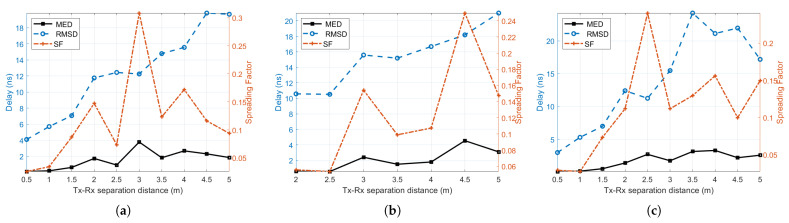
Time dispersion parameters in (**a**) the Manufacturing hall at Dokka Fastners AS, (**b**) the Test room at Dokka Fastners AS, and (**c**) NTNU MANULAB. MED, mean excess delay.

The time dispersion parameters along the Tx-Rx separation distance at the three considered industrial environments are presented in Figure 10a–c. From visual inspections of the figures, it can be shown that the RMSDS increases as the Tx-Rx separation distance increases. The RMSDS values vary from 4.1 ns to 19.8 ns, from 10.6 ns to 21.0 ns, and from 3.0 ns to 24.2 ns for the Manufacturing hall at Dokka Fastners AS, the Test room at Dokka Fastners AS, and the NTNU MANULAB environments, respectively. The mean excess delay is less than 5 ns at all locations, as can be seen by the black curve plots in Figure 10a–c. It can be observed that the spreading factor of (8) is less than unity at all locations along with the Tx-Rx separation distances, implying that most of the MPCs fall in the early delay bins with a high power concentration. The minimum, maximum, and average values of RMSDS are listed in Table 5.

### 6.5. Results Comparison

In this section, the results of path loss and RMSDS are compared with the results of the previous studies reviewed in Section 2.3 and Section 3. However, due to the inherent differences in the measured environment and the measurement and modeling methodology, e.g., the frequency and the range of measurements, the path loss and RMSDS may not be directly comparable. Hence, our approach cannot be generalized to all environments and frequencies. However, the effects of the environment on the channel characteristics can be observed from the similarities and contrasts in different propagation scenarios at different frequencies. The PLE values for our measurements and the previous conducted measurements in different indoor offices at 140 GHz [8,33,34,35] are comparable and close to the free space PLE. For comparison with industrial measurements in the mmWave band below 100 GHz, the PLE values of 1.9 at 28 GHz [50] and at 60 GHz [44] in industrial environments are almost identical to our measurements.

The studies of RMSDS at frequency bands above 100 GHz are very limited. It can be noted that the RMSDS values at indoor offices are less than 0.5 ns at 310 GHz and 140 GHz [30,33], which are very low compared to our measured RMSDS. For measurements in the mmWave band at 28 GHz in industrial environments [50], the RMSDS was 23.1 ns, which is comparable with our maximum value of RMSDS. In [44], it was also found that the RMSDS mean values in light industrial environment were 13.7 ns and 13.4 ns at 28 GHz and 60 GHz, respectively, which are almost identical to our mean value of RMSDS at NTNU MANULAB.

## 7. Conclusions

This paper investigates the radio channel propagation characteristics in the 107–109 GHz band in three different industrial environments. An overview of the radio propagation studies in the frequency band above 100 GHz for 6G was presented. We also gave an overview of radio propagation studies in indoor industrial environments. Based on wideband measurements conducted in three different industrial settings, several radio channel propagation characteristics, such as the path loss, K-factor, time dispersion, and multipath propagation, were investigated. The CI and FI path loss models were used to examine the large-scale fading. The PLE values were less than the FSPL exponent, indicating that the reflected paths contribute additional energy to the received signal from the direct path. The high K-factor values were observed at the short Tx-Rx separation distances below 2 m in all industrial environments. The RMSDS values ranged from 3 ns to 24 ns, which were more than the MEDs and resulted in a spreading factor value of less than unity. This means that most of the high power MPCs fall in the early delay bins of the PDP. The findings from this work with the extensive overview of the radio propagation studies in 6G networks, as well as the measurement results of 107–109 GHz in three different industrial environments can help engineers and researchers to plan, design, and optimize reliable 6G wireless indoor networks.

## Figures and Tables

**Table 1:** Channel characterization studies in the 0.1–1 THz range. RMSDS, root mean squared delay spread.

Reference	Frequency (GHz)	Scenario	Link Length (m)	Channel Characteristics
[14]	100–300	Outdoor LoS	8	Snow attenuation
[15]	103	Outdoor LoS	400	Rain attenuation
[16,17]	120	Outdoor LoS	400	Rain attenuation
[18]	140.7	Outdoor LoS	800	Rain attenuation
[19]	148 and 156	Outdoor LoS	325	Rain attenuation
[9,27]	300	Indoor LoS Indoor NLoS	1.67 4.7	Path loss, RMSDS, and reflection
[26]	300	Indoor room	-	diffraction
[28,29]	300	Indoor office	-	Path loss, K-factor, RMSDS, and AoA spread
[30]	300–320	Indoor desktop LoS Indoor desktop NLoS	0.7 0.3	Path loss and RMSDS
[31]	300–316	Indoor desktop LoS	0.25	Path loss
[37]	350	Indoor LoS	8 and 1	Path loss and throughput
[32]	260–400	Indoor desktop LoS Indoor desktop NLoS	0.1–0.95 0.1–0.35	Path loss and throughput
[25]	100	Typical building materials	-	Effective attenuation
[33]	140	Indoor LoS, OLoS, and NLoS	0.4–0.9	Path loss, reflection, and RMSDS
[34]	140	Indoor LoS shopping mall	35	Path loss
[8]	140	Indoor office LoS	5	Path loss
[35]	126–156	Indoor office LoS	1.6	Path loss

**Table 2:** Channel characterization studies in indoor industrial environments in the mmWave band.

Reference	Frequency (GHz)	Scenario	Link Length (m)	Channel Characteristics
[46]	28	Factory LoS and NLoS	100	Path loss
[47]	28	Engineering workshop	-	Reflection
[49]	28	Circle-shaped machine hall LoS and NLoS	11.5–42	Path loss and RMSDS
[50]	28	Circle-shaped machine hall LoS and NLoS	4–26	Path loss and RMSDS
[51]	57–62	Metal cabinet	0.25–2.5	Path loss and RMSDS
[44]	28 and 60	Factory LoS and NLoS	35	Path loss and RMSDS
[52]	60	LoS and NLoS in different industrial settings	10–100	Throughput

**Table 3:** Measurement setup parameters. HPBW, half power beam-width.

Parameter	Value
Frequency (GHz)	108
Bandwidth (GHz)	2
Delay Resolution (ns)	0.5
Tx Power (dBm)	0
Tx Antenna Type	Horn
Rx Antenna Type	Horn
Tx/Rx Polarization	Vertical/vertical
Tx/Rx Antenna Gain (dBi)	21.14/21.14
Tx/Rx Antenna Azimuth HPBW	$15.7^{\circ }$/$15.7^{\circ }$
Tx/Rx Antenna Height (m)	1.6/1.6

**Table 4:** Path loss models’ parameters. PLE, path loss exponent.

Environment	Model	PLE for CI β for FI	FI (α) (dB)	σ (dB)
Manufacturing hall at Dokka Fastners AS	CI	1.7	-	0.4
	FI	1.7	73.3	0.4
Test room at Dokka Fastners AS	CI	1.6	-	0.3
	FI	1.6	73.1	0.3
NTNU MANULAB	CI	1.9	-	0.7
	FI	2	71.5	0.7

**Table 5:** K-factor and RMSD at all measurement locations.

Environment	K-Factor (dB) (Min., Max., Avg)	RMSDS (ns) (Min., Max., Avg.)
Manufacturing hall at Dokka Fastners AS	1.8, 21.6, 9.6	4.1, 19.8, 12.3
Test room at Dokka Fastners AS	0.9, 19.9, 8.8	10.5, 21.0, 15.4
NTNU MANULAB	−0.3, 25.9, 9.9	3.0, 24.2, 13.9

## Data Availability

Data available on request from the authors.
